# Ultrasound-Guided Humerus-Parallel Injectate Distribution to the Posterior Antebrachial Cutaneous Nerve-Related Fascial Plane and Common Extensor Origin: A Proof-of-Concept Cadaveric Anatomical Feasibility Study

**DOI:** 10.3390/diagnostics16111698

**Published:** 2026-05-31

**Authors:** Sang-Hyun Kim, U-Young Lee, Yonghyun Yoon, Seungbeom Kim, Dongyeun Sung, Jungyoun Kim, Seunguk Lee, Ki-Tae Kim, King Hei Stanley Lam

**Affiliations:** 1 College of Korean Medicine, Woosuk University, Wanju-Gun 55338, Republic of Korea; amalang@naver.com; 2 Department of Anatomy, Catholic Institute for Applied Anatomy, College of Medicine, The Catholic University of Republic of Korea, Seoul 06591, Republic of Korea; 3 Department of Orthopaedic Surgery, Gangnam Sacred Heart Hospital, Hallym University College of Medicine, Seoul 07441, Republic of Korea; 4 Incheon Terminal Orthopedic Surgery Clinic, Incheon 21574, Republic of Korea; 5 International Academy of Regenerative Medicine, Incheon 21574, Republic of Korea; 6 Board of Clinical Research, The International Association of Musculoskeletal Medicine, Hong Kong, China; 7 MSKUS, 1035 E. Vista Way #128, Vista, CA 92084, USA; 8 Miso Pain Clinic, 1569, Bongyeong-Ro, Yeongtong-Gu, Suwon-Si 16703, Republic of Korea; 9 Himchannamu Neurosurgery Clinic, Daegu 41423, Republic of Korea; 10 Faculty of Medicine, The University of Hong Kong, Hong Kong, China; 11 Faculty of Medicine, The Chinese University of Hong Kong, Hong Kong, China

**Keywords:** tennis elbow, ultrasonography, interventional, posterior antebrachial cutaneous nerve, elbow joint, cadaver, peripheral nerves, injections, fascia

## Abstract

**Background:** Lateral epicondylopathy is commonly approached as a tendinopathic disorder of the common extensor origin; however, persistent lateral elbow pain may also involve a superficial sensory nerve component related to the posterior antebrachial cutaneous nerve (PABCN). This proof-of-concept cadaveric anatomical feasibility study evaluated whether a single-window, humerus-parallel ultrasound-guided injectate pathway could simultaneously reach the superficial PABCN-related fascial plane and the common extensor origin. **Methods:** One fresh-frozen male cadaveric donor was used, and both elbows were injected under real-time ultrasound guidance. With the elbow flexed and the forearm pronated, the transducer was aligned parallel to the long axis of the humerus over the lateral epicondylar region. A 23-gauge, 6 cm needle was advanced in plane from distal to proximal over the common extensor aponeurosis, and 10 mL of 1% methylene blue was injected into each elbow. Layer-by-layer anatomical dissection was then performed by an anatomist who was not involved in the injection procedure. Gross linear dye spread was measured directly during dissection using the distal needle entry point as the reference point, and ruler-containing photographs were additionally reviewed using ImageJ software for supportive image-assisted assessment. **Results:** In both elbows, methylene blue stained the superficial PABCN-related fascial plane, including the anterior and posterior branches of the PABCN, and concomitantly covered the common extensor aponeurosis and lateral epicondylar enthesis. Dye spread measured approximately 10 cm proximally, 5 cm distally, and 4 cm anteriorly. No gross intra-articular dye deposition or focal intramuscular pooling was observed. **Conclusions:** This proof-of-concept cadaveric study demonstrates the anatomical plausibility of a single-window, enthesis-centered ultrasound-guided injectate pathway that includes both the superficial PABCN-related plane and the common extensor origin. These findings should be interpreted as descriptive anatomical feasibility observations and do not establish reproducibility across anatomical variants, clinical efficacy, safety, or procedural superiority.

## 1. Introduction

Lateral epicondylopathy is one of the most common causes of lateral elbow pain and is typically attributed to degenerative pathology of the common extensor origin, particularly the extensor carpi radialis brevis [1]. Although the condition has historically been described as “lateral epicondylitis,” the underlying pathology is now more commonly understood as a tendinopathic process rather than a purely inflammatory disorder. For this reason, many diagnostic and therapeutic approaches have focused on the tendon–enthesis complex at the lateral epicondyle, including rehabilitation, image-guided injections, and surgical procedures in refractory cases.

However, persistent or recurrent lateral elbow pain may not always be explained by common extensor tendinopathy alone. Several anatomic and clinical pain generators may coexist around the lateral elbow, including posterior interosseous nerve compression, synovial plica-related pain, and involvement of superficial sensory nerve branches [2,3,4]. Among these structures, the posterior antebrachial cutaneous nerve (PABCN), also termed the posterior cutaneous nerve of the forearm (PCNF), is particularly relevant because of its superficial course near the lateral epicondylar region and its potential contribution to chronic lateral elbow pain [5,6]. Prior anatomical and sonographic studies have demonstrated that the PABCN and its distal branches can be identified using high-resolution ultrasound, and PABCN-centered perineural injection techniques have been described in both cadaveric and clinical contexts [7].

The potential role of the PABCN in chronic lateral elbow pain has led to increasing interest in ultrasound-guided diagnostic and therapeutic interventions directed at this nerve. Perineural injection of the posterior cutaneous nerve of the forearm has been reported as a potential diagnostic and therapeutic option for chronic lateral elbow pain, and radiofrequency ablation of an epicondylar branch has also been described in refractory lateral epicondylosis [7,8]. These reports suggest that a neural component may be present in selected patients with recalcitrant lateral epicondylopathy. Nevertheless, previously described approaches have generally been nerve-centered or tendon-centered rather than designed to address both the superficial PABCN-related fascial plane and the common extensor origin–lateral epicondylar enthesis complex within a single integrated procedural window. To our knowledge, no prior cadaveric anatomical study has demonstrated a single-window, single-pathway ultrasound-guided injection approach capable of concurrently reaching both the superficial PABCN-related fascial plane and the common extensor enthesis.

This distinction is clinically important because lateral epicondylopathy may represent a mixed pain state in which tendinous, entheseal, and neural structures contribute simultaneously to symptoms. A procedure that targets only the common extensor origin may fail to address a superficial sensory nerve component, whereas a purely perineural injection may not adequately cover the tendon–enthesis complex. Therefore, an anatomically defined ultrasound-guided pathway capable of accessing both the PABCN-related fascial plane and the common extensor origin may provide a useful technical foundation for future diagnostic and interventional studies. However, such a cadaveric anatomical demonstration should not be interpreted as evidence of clinical efficacy, procedural superiority, or reproducibility across anatomical variants.

To address this need, we developed an ultrasound-guided humerus-parallel longitudinal injectate-distribution approach along the intended fascial plane. In this approach, both the transducer and needle trajectory are oriented parallel to the long axis of the humerus, and a posterior-to-anterior sonographic sweep is used to define a lateral epicondylar working window. The needle is then advanced in plane from distal to proximal over the common extensor aponeurosis, with the aim of allowing injectate to distribute along the superficial PABCN-related fascial plane while also covering the common extensor aponeurosis and lateral epicondylar enthesis. The novelty of this study is not the sonographic identification of the PABCN itself, but the cadaveric demonstration of a single enthesis-centered fascial-plane pathway that anatomically encompasses both superficial sensory nerve-related branches and the common extensor tendon–enthesis target region. The purpose of this proof-of-concept cadaveric anatomical feasibility study was to evaluate whether this humerus-parallel ultrasound-guided pathway could achieve simultaneous methylene blue distribution to the PABCN-related superficial fascial plane and the common extensor origin in a fresh-frozen cadaveric model. This study was designed to assess anatomical target accessibility and injectate distribution, not clinical efficacy, procedural superiority, or anatomical variability across donors.

## 2. Materials and Methods

### 2.1. Study Design and Ethical Considerations

This study was designed as a proof-of-concept cadaveric anatomical feasibility study to evaluate whether an ultrasound-guided humerus-parallel longitudinal injectate-distribution pathway could deliver methylene blue simultaneously to the superficial posterior antebrachial cutaneous nerve (PABCN)-related fascial plane and the common extensor origin in the lateral elbow.

The study focused on anatomical dye distribution after ultrasound-guided injection rather than clinical efficacy. Therefore, no patient outcomes, therapeutic responses, or comparative statistical analyses were assessed. Because both elbows were obtained from a single cadaveric donor, the two elbows were regarded as descriptive procedural replications rather than independent biological samples. Accordingly, the unit of technical assessment was the ultrasound-guided injection pathway and its gross anatomical dye distribution, not interindividual anatomical variability. The study was not designed to assess procedural reproducibility across donors, anatomical variation in PABCN branching patterns, operator-independent feasibility, clinical efficacy, or superiority over existing injection techniques.

The cadaveric protocol was reviewed by the Institutional Review Board of the Catholic University of Korea and was granted exemption from full ethical review because the study involved cadaveric specimens only and did not include living participants in the cadaveric experiment or identifiable personal data (IRB No. MIRB-면20260120-006; 20 January 2026).

**Figure 1:**
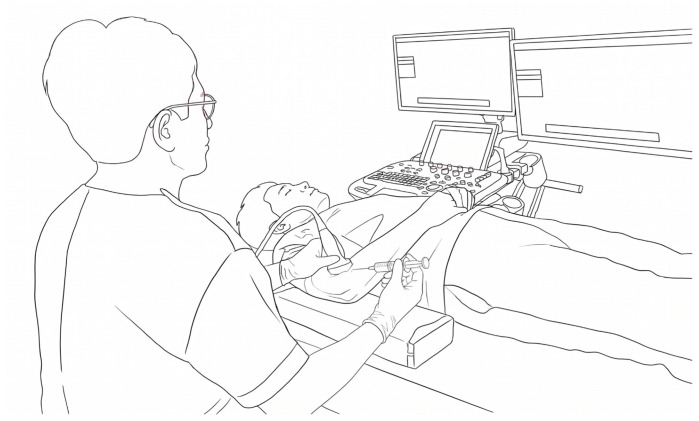
Volunteer positioning and humerus-parallel transducer orientation for the lateral elbow approach.

**Figure 2:**
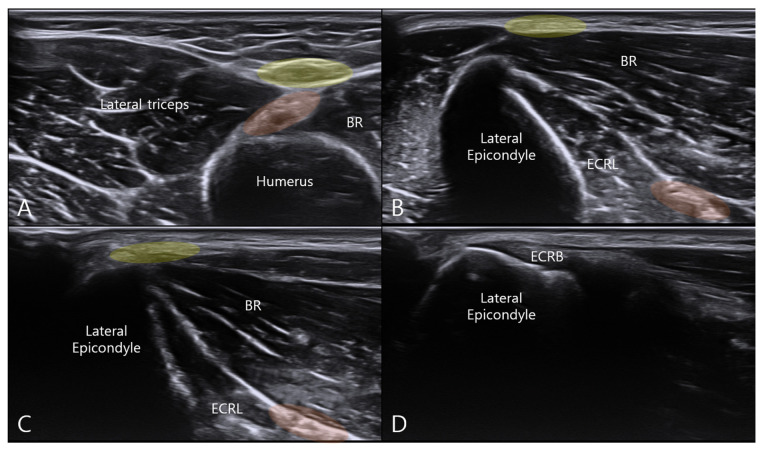
Predefined sonographic orientation sequence for tracing the posterior antebrachial cutaneous nerve from the distal spiral groove to the lateral epicondylar region. (**A**) At the distal spiral groove region, the PABCN is identified in relation to the humerus, lateral triceps, and brachioradialis. (**B**) With distal probe translation, the PABCN is visualized in relation to the brachioradialis (BR) and extensor carpi radialis longus (ECRL). (**C**) Further distal tracing demonstrates the PABCN approaching the lateral epicondylar region. (**D**) The final transducer position demonstrates the relationship between the PABCN course and the lateral epicondylar region, where the extensor carpi radialis brevis (ECRB) is visualized near the lateral epicondyle. Yellow overlay indicates the PABCN. Orange overlay indicates the proximal nerve segment used for anatomical orientation. BR, brachioradialis; ECRL, extensor carpi radialis longus; ECRB, extensor carpi radialis brevis. These images were obtained from a healthy volunteer for illustrative anatomical orientation only. This tracing sequence was not used as a comparative injection arm and was not analyzed for procedural time, accuracy, or superiority.

**Figure 3:**
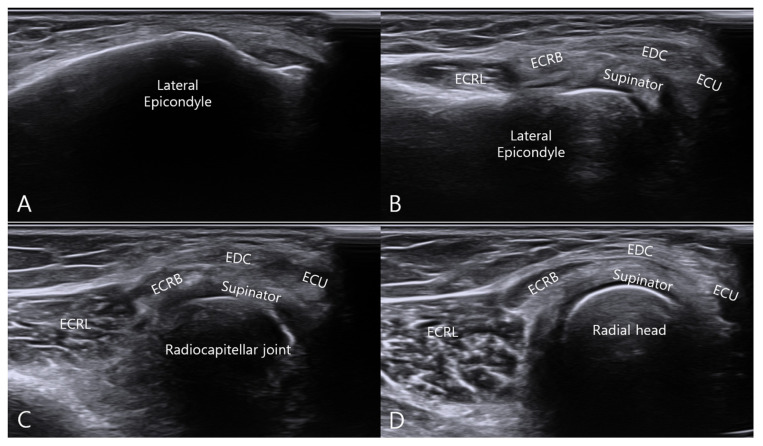
Humerus-parallel posterior-to-anterior sonographic sweep defining the single-window lateral elbow approach. The transducer was aligned parallel to the long axis of the humerus over the lateral epicondylar region and swept from posterior to anterior to define the proposed humerus-parallel longitudinal working window. This predefined sweep sequentially demonstrated: (**A**) the lateral epicondyle; (**B**) the lateral ligament–common extensor tendon enthesis complex, including the extensor carpi radialis longus (ECRL), extensor carpi radialis brevis (ECRB), extensor digitorum communis (EDC), extensor carpi ulnaris (ECU), and supinator; (**C**) the radiocapitellar joint region; and (**D**) the radial head region. This posterior-to-anterior sweep was used to identify the intended superficial fascial-plane corridor overlying the common extensor origin within a single humerus-parallel longitudinal transducer orientation. ECRL, extensor carpi radialis longus; ECRB, extensor carpi radialis brevis; EDC, extensor digitorum communis; ECU, extensor carpi ulnaris.

Representative positioning and sonographic tracking images shown in Figure 1, Figure 2 and Figure 3 were obtained from a healthy volunteer for procedural illustration only. Written informed consent for image acquisition and publication was obtained from the volunteer.

### 2.2. Cadaver Preparation

The injection experiment was performed using one fresh-frozen, non-embalmed male cadaveric specimen (age, 76 years; height, 160 cm; weight, 54 kg). The specimen was thawed at room temperature prior to the procedure to approximate physiological tissue conditions. Both elbows from the same donor were used for the ultrasound-guided injection procedure and subsequent anatomical dissection.

Before injection, complete thawing was confirmed by manual palpation and gross inspection of the elbow region, and no embalming-related tissue stiffness or fixation artifact was present. The skin, subcutaneous tissues, superficial fascial planes, PABCN, common extensor aponeurosis, and lateral epicondylar enthesis were preserved for procedural assessment. No gross structural disruption, prior surgical alteration, or severe deformity was identified in the lateral elbow region that would preclude standardized ultrasound-guided injection or anatomical dissection.

Because cadaveric tissue lacks physiological perfusion and dynamic tissue compliance, the observed dye distribution was interpreted as an anatomical demonstration of the intended injectate pathway rather than a direct simulation of in vivo hydrodissection behavior.

### 2.3. Cadaveric Positioning

The cadaveric specimen was placed in the supine position. The shoulder was positioned in slight adduction and internal rotation, with the elbow flexed to approximately 90° and the forearm pronated so that the hand rested on the abdomen. This configuration resembles the arm position used during the belly press or Napoleon test and provided a clinically familiar, stable posture for lateral elbow exposure.

A small bolster was placed beneath the medial aspect of the elbow to elevate and stabilize the elbow, thereby improving access to the lateral epicondylar region during ultrasound scanning and needle advancement. This positioning facilitated sonographic visualization of the lateral epicondyle, common extensor origin, radiocapitellar region, and superficial fascial plane containing the posterior antebrachial cutaneous nerve (PABCN) branches.

For procedural illustration, the corresponding limb position and operator setup in a healthy volunteer are shown in Figure 1; the volunteer images were used only to demonstrate positioning and sonographic workflow and were not part of the cadaveric injection experiment.

The healthy volunteer was positioned supine with the shoulder in slight adduction and internal rotation, the elbow flexed to approximately 90°, and the forearm pronated so that the hand rested on the abdomen. A small bolster was placed beneath the medial aspect of the elbow to elevate and stabilize the lateral elbow region. The transducer was aligned parallel to the long axis of the humerus over the lateral epicondylar region, corresponding to the proposed humerus-parallel longitudinal working window. This figure was obtained from a healthy volunteer to illustrate positioning and sonographic workflow only and was not part of the cadaveric injection experiment. Written informed consent was obtained from the volunteer for image acquisition and publication.

### 2.4. Ultrasound System and Imaging Parameters

Ultrasonographic evaluation and injection were performed using an Alpinion XC90 Elite ultrasound system (ALPINION MEDICAL SYSTEMS Co., Ltd., Seoul, Republic of Korea) equipped with a linear transducer. The transducer had a center frequency of 7 MHz and an operating range of 3–19 MHz. Before needle insertion, the anticipated needle path was assessed using grayscale ultrasound, and color Doppler imaging was used when necessary to identify superficial vessels or vascular variants within the planned humerus-parallel working window.

The imaging depth was set at 3 cm, and the dynamic range was set at 60 dB. Coupling gel was applied to optimize acoustic transmission. All ultrasound examinations and injections were performed by a single orthopedic surgeon with more than 10 years of experience in musculoskeletal ultrasound-guided procedures.

### 2.5. Sonographic Tracking Protocol

For anatomical orientation and procedural standardization, a predefined sonographic scanning sequence was used before injection. The transducer was initially placed at the distal spiral groove region to identify the proximal course of the PABCN using the overlay-guided sonographic orientation image (Figure 2A). The PABCN was then traced distally in relation to the brachioradialis and extensor carpi radialis longus (Figure 2B). With further distal translation of the probe, the PABCN was observed to course closer to the lateral epicondylar region and the bony surface (Figure 2C). Final distal tracing allowed localization of the lateral epicondylar enthesis in relation to the PABCN trajectory (Figure 2D). This distal spiral groove-based tracking sequence was used only for anatomical orientation and to confirm the expected course of the PABCN before applying the proposed humerus-parallel working window. It was not used as a comparative injection arm and was not analyzed for procedural time, accuracy, or superiority.

The posterior antebrachial cutaneous nerve (PABCN), also termed the posterior cutaneous nerve of the forearm, was traced in a healthy volunteer for anatomical orientation before defining the proposed humerus-parallel working window.

In the proposed approach, the transducer was aligned parallel to the long axis of the humerus over the lateral epicondylar region. With the limb positioned as described above, the transducer was swept from posterior to anterior across the lateral elbow to define a humerus-parallel longitudinal working window. This sweep sequentially demonstrated the lateral epicondyle (Figure 3A), the lateral ligament–common extensor tendon enthesis complex (Figure 3B), the radiocapitellar joint (Figure 3C), and the radial head (Figure 3D). This window was intended to provide a single ultrasound-guided procedural field for accessing both the superficial PABCN-related fascial plane and the common extensor aponeurosis–enthesis complex.

### 2.6. Ultrasound-Guided Injection Procedure

**Figure 4:**
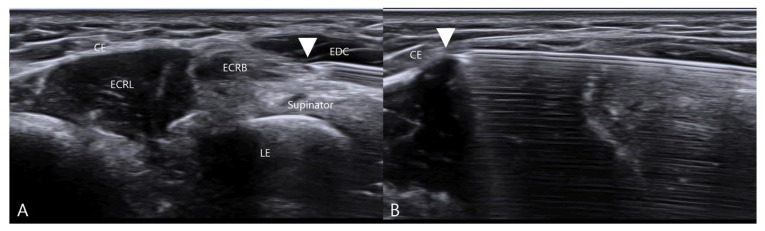
Real-time ultrasound-guided distal-to-proximal in-plane needle advancement and injectate delivery. (**A**) A 23-gauge, 6 cm needle was introduced from a distal skin entry point under real-time in-plane ultrasound guidance. The needle was oriented proximally and maintained nearly parallel to the skin surface and the long axis of the humerus. (**B**) The needle tip was advanced to the superficial fascial plane overlying the common extensor aponeurosis (CE) under continuous sonographic visualization before injectate delivery. The intended target plane was located superficial to the CE and adjacent to the PABCN-related fascial plane. Arrowhead, needle tip; CE, common extensor aponeurosis; LE, lateral epicondyle; ECRL, extensor carpi radialis longus; ECRB, extensor carpi radialis brevis; EDC, extensor digitorum communis.

Under real-time ultrasound guidance, a 23-gauge, 6 cm needle was introduced from a distal skin entry point using an in-plane technique (Figure 4A). The needle was advanced from distal to proximal, with the needle shaft maintained as parallel as possible to the skin surface and the long axis of the humerus. This trajectory was selected to allow continuous in-plane visualization of the needle shaft and tip while advancing over the superficial aspect of the common extensor aponeurosis and toward the intended superficial fascial plane.

The needle tip was directed toward the fascial plane overlying the common extensor aponeurosis and lateral epicondylar origin. After confirmation of needle tip position, the needle was advanced over the extensor aponeurosis under continuous in-plane visualization (Figure 4B). A total of 10 mL of 1% methylene blue was injected slowly into each elbow. This volume was selected not to simulate a selective PABCN block, but to visualize the potential distribution of injectate along a broader superficial fascial plane intended to include both the PABCN-related plane and the common extensor origin. During injection, the transducer was adjusted as needed to monitor longitudinal spread across the intended target region. Slow injectate delivery was used to reduce superficial tissue distention, maintain probe stability, and preserve visualization of the needle tip and expanding fluid plane. Throughout the needle advancement and injection, the needle tip was kept under continuous sonographic visualization. Injection was performed slowly and with low manual pressure, and the needle trajectory was adjusted if the needle tip approached an unintended deep tissue plane, joint space, or visible vascular structure.

During injection, slow low-pressure delivery of 10 mL of 1% methylene blue produced longitudinal fluid expansion along the superficial plane rather than focal subcutaneous pooling. The corresponding real-time ultrasound-guided needle advancement and injectate delivery sequence is provided in Supplementary Video S1.

Methylene blue was selected as a visual tracer to allow direct identification of injectate distribution during subsequent anatomical dissection. Visual tracer injection followed by layer-by-layer dissection has been widely used in cadaveric validation studies to confirm whether an ultrasound-guided target or fascial plane can be reached anatomically [7,9,10]. In the present study, methylene blue was used to assess gross dye distribution along the intended superficial fascial plane rather than to reproduce the behavior of a specific clinical injectate. Because methylene blue has lower viscosity and potentially greater diffusion than many clinical injectates, including platelet-rich plasma or other biologic preparations, the observed dye distribution should be interpreted as an anatomical feasibility finding rather than a direct simulation of in vivo injectate behavior or confirmation of true mechanical hydrodissection. The real-time ultrasound-guided injection sequence is provided as Supplementary Video S1.

### 2.7. Anatomical Dissection and Outcome Assessment

Approximately 1 h after injection, layer-by-layer anatomical dissection was performed by an experienced anatomist who was not involved in the ultrasound-guided injection procedure. The injection operator and the anatomist performed their respective procedures independently. Before dissection, the anatomist was not informed of the detailed needle trajectory, intended direction of injectate spread, or anticipated target distribution. The skin and subcutaneous tissues were carefully reflected to preserve the PABCN, its anterior and posterior branches, the common extensor aponeurosis, and the lateral epicondylar enthesis. For this gross anatomical assessment, positive staining was defined as any visible methylene blue discoloration of the dissected fascial plane or target structure, including faint blue discoloration. This definition was selected to determine whether the intended anatomical plane had been reached, not to quantify dye concentration or staining intensity.

The primary outcome was visual confirmation of methylene blue spread along the PABCN-related fascial plane and staining of the anterior and posterior branches of the PABCN. Additional assessment focused on whether the dye reached the common extensor aponeurosis and lateral epicondylar enthesis. Successful dual-target dye distribution was defined as gross staining of both (1) the superficial PABCN-related fascial plane, including the PABCN or at least one identifiable PABCN branch, and (2) the common extensor aponeurosis or lateral epicondylar enthesis on anatomical dissection. Unsuccessful dual-target distribution was defined as isolated tendon–enthesis staining without staining of the PABCN-related superficial fascial plane, isolated PABCN-related plane staining without coverage of the common extensor origin, focal subcutaneous or intramuscular pooling without fascial-plane spread, or gross intra-articular dye deposition.

Secondary observational outcomes included the proximal, distal, and anterior linear extents of dye spread from the distal needle entry point. Linear dye spread was measured directly during anatomical dissection by the anatomist using a metric ruler. The distal needle entry point was used as the reference point for all measurements, and the proximal, distal, and anterior extents of visible dye spread were recorded. The ruler was placed along the posterior aspect of the elbow during measurement to document the gross anatomical extent of dye distribution. Available ruler-containing photographs were additionally reviewed using ImageJ software (version 1.54p; National Institutes of Health, Bethesda, MD, USA) for calibrated image-assisted confirmation of the linear spread distances. The visible metric ruler in each photograph was used for pixel-to-centimeter calibration, and the distal needle entry point was used as the reference point. ImageJ-assisted measurements were consistent with the direct gross anatomical measurements of approximately 10 cm proximally, 5 cm distally, and 4 cm anteriorly. Because the photographs were obtained for anatomical documentation rather than under a fully standardized quantitative imaging protocol, ImageJ review was used only as supportive image-assisted assessment of gross linear measurements. No formal segmentation of stained versus unstained areas, volumetric quantification, proportional coverage analysis, dye-intensity analysis, histologic validation, or statistical comparison was performed.

## 3. Results

### 3.1. Procedural Feasibility of the Ultrasound-Guided Approach

Ultrasound-guided injections were completed in both elbows of the fresh-frozen cadaveric specimen using the predefined humerus-parallel longitudinal protocol. In both elbows, the lateral epicondyle, common extensor aponeurosis, radiocapitellar region, and superficial fascial plane overlying the common extensor origin were visualized within the same humerus-parallel longitudinal working window.

The posterior-to-anterior sonographic sweep allowed identification of the intended injection corridor within the lateral epicondylar region. Long-segment distal tracing of the PABCN from the spiral groove was used only for anatomical orientation and was not required during the actual cadaveric injection procedure. The distal-to-proximal in-plane needle trajectory enabled continuous visualization of the needle shaft and tip as the needle advanced over the common extensor aponeurosis. No gross procedural difficulty, unintended deep penetration, or disruption of the target tissue plane was observed during either injection.

### 3.2. Real-Time Injectate Delivery

During injection, real-time ultrasound demonstrated longitudinal fluid expansion along the superficial fascial plane overlying the common extensor origin in both elbows. The injectate distributed along the intended superficial plane rather than forming a focal subcutaneous bleb. The expanding fluid plane extended across the lateral epicondylar region and remained superficial to the common extensor aponeurosis during real-time sonographic monitoring. The injection was completed without sonographic evidence of unintended intra-articular injection or focal intramuscular deposition.

### 3.3. Gross Anatomical Dye Distribution

**Figure 5:**
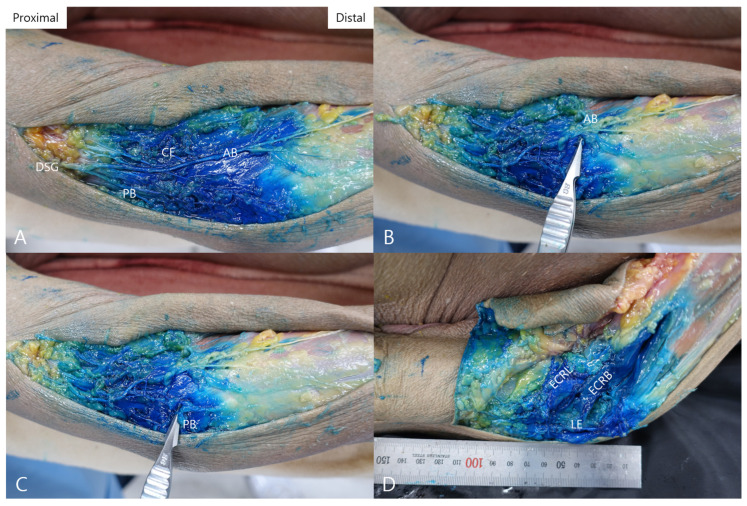
Cadaveric dye distribution and gross linear measurement after humerus-parallel ultrasound-guided injectate delivery. Layer-by-layer anatomical dissection was performed approximately 1 h after injection by an experienced anatomist who was not involved in the ultrasound-guided injection procedure. (**A**) After skin removal, methylene blue is visible over the lateral elbow region, and the posterior antebrachial cutaneous nerve (PABCN) is identified within the stained superficial fascial plane. (**B**) Dissection of the anterior branch (AB) of the PABCN demonstrates methylene blue staining along its course. (**C**) The posterior branch (PB) of the PABCN is similarly stained, indicating that the injectate reached both identifiable PABCN branches. (**D**) A metric ruler was placed along the posterior aspect of the elbow during anatomical dissection to document the gross linear extent of dye distribution from the distal needle entry point. Direct ruler measurement demonstrated dye distribution of approximately 10 cm proximally, 5 cm distally, and 4 cm anteriorly. The ruler-containing photograph was additionally reviewed using ImageJ software (version 1.54p; National Institutes of Health, Bethesda, MD, USA) for calibrated image-assisted assessment of these linear spread distances. Dye staining was also observed over the lateral epicondylar enthesis (LE) and the overlying common extensor aponeurosis, indicating gross anatomical coverage of both the superficial PABCN-related fascial plane and the enthesis-centered target region. Visible staining was defined as any methylene blue discoloration of the dissected fascial plane or target structure, including faint blue discoloration. ImageJ review was used only as supportive image-assisted assessment of gross linear measurements. No formal segmentation of stained versus unstained areas, volumetric quantification, proportional coverage analysis, dye-intensity analysis, or histologic validation was performed. PABCN, posterior antebrachial cutaneous nerve; AB, anterior branch; PB, posterior branch; DSG, distal spiral groove; LE, lateral epicondylar enthesis; CE, common extensor aponeurosis; ECRB, extensor carpi radialis brevis; ECRL, extensor carpi radialis longus.

Layer-by-layer anatomical dissection demonstrated methylene blue distribution along the superficial PABCN-related fascial plane of the lateral elbow in both injected elbows. After removal of the skin and superficial subcutaneous tissue, dye was visible over the lateral epicondylar region, and the PABCN was identified within the stained superficial tissue plane (Figure 5A). Both the anterior and posterior branches of the PABCN showed dye staining, indicating that the injectate reached the superficial PABCN-related plane rather than remaining limited to the tendon surface alone (Figure 5B,C). Dye also extended proximally toward the distal spiral groove region, supporting gross anatomical continuity of the injected superficial fascial plane.

### 3.4. Coverage of the Common Extensor Origin and Lateral Epicondylar Enthesis

Gross dissection confirmed methylene blue coverage of the common extensor aponeurosis and lateral epicondylar enthesis in both elbows. The dye was observed along the superficial surface of the common extensor origin and extended across the enthesis-centered target region (Figure 5D). Together with the PABCN branch staining described above, this finding indicated simultaneous gross dye distribution to two anatomically relevant regions: the superficial PABCN-related fascial plane and the common extensor aponeurosis–enthesis complex. This dual-target distribution pattern was observed in both elbows using the same predefined injection protocol.

### 3.5. Descriptive Spatial Extent of Dye Spread

The spatial extent of dye distribution was assessed by direct gross anatomical measurement during dissection. The distal needle entry point was used as the reference point, and the proximal, distal, and anterior extents of visible methylene blue staining were measured by the anatomist using a metric ruler. Visible staining was defined as any blue discoloration of the dissected fascial plane or target structure, including faint methylene blue discoloration.

From the distal needle entry point, dye distribution measured approximately 10 cm proximally, 5 cm distally, and 4 cm anteriorly across the lateral elbow soft-tissue plane. These direct gross anatomical measurements were additionally reviewed using ruler-containing photographs and ImageJ software (version 1.54p; National Institutes of Health, Bethesda, MD, USA). The visible metric ruler in each photograph was used for pixel-to-centimeter calibration, and the distal needle entry point was used as the reference point. ImageJ-assisted measurements were consistent with the direct gross anatomical measurements.

The stained region included the lateral epicondylar enthesis, common extensor aponeurosis, and superficial PABCN-related fascial plane. Because the photographs were obtained for anatomical documentation rather than under a fully standardized quantitative imaging protocol, ImageJ review was used only as supportive image-assisted assessment of gross linear measurements. No formal segmentation of stained versus unstained areas, volumetric quantification, proportional coverage analysis, dye-intensity analysis, histologic validation, or statistical comparison was performed. The procedural and anatomical findings are summarized in Table 1.

## 4. Discussion

This proof-of-concept cadaveric anatomical feasibility study evaluated a humerus-parallel ultrasound-guided injectate-distribution pathway designed to access both the superficial posterior antebrachial cutaneous nerve (PABCN)-related fascial plane and the common extensor origin in the lateral elbow. The principal finding was that methylene blue delivered through a distal-to-proximal in-plane trajectory over the common extensor aponeurosis stained both the anterior and posterior branches of the PABCN while also covering the common extensor aponeurosis and lateral epicondylar enthesis in both elbows of a fresh-frozen cadaveric donor. Direct gross anatomical measurement, supported by calibrated ImageJ-assisted review of ruler-containing photographs, showed dye distribution of approximately 10 cm proximally, 5 cm distally, and 4 cm anteriorly from the distal needle entry point. These findings support the anatomical plausibility of a single enthesis-centered fascial-plane pathway for dual-region dye distribution, while not establishing clinical efficacy, procedural superiority, or reproducibility across anatomical variants.

Lateral epicondylopathy is commonly conceptualized as a tendinopathic disorder of the common extensor origin, particularly involving the extensor carpi radialis brevis. Accordingly, many image-guided interventions have focused on the tendon substance or the lateral epicondylar enthesis. However, chronic lateral elbow pain may not always be explained by tendinopathy alone. Coexisting pain generators, including posterior interosseous nerve irritation, synovial plica-related pain, radiocapitellar pathology, and superficial sensory nerve involvement, may contribute to persistent symptoms in selected patients [4]. In this context, the PABCN is anatomically relevant because it courses superficially around the posterolateral elbow and gives rise to distal branches that may overlap the region in which patients with lateral epicondylopathy commonly report pain.

Previous anatomical and sonographic studies have shown that the posterior cutaneous nerve of the forearm and its distal branches can be visualized with high-resolution ultrasound [6,7,11]. PABCN-centered perineural injection has also been proposed as a diagnostic and potentially therapeutic option in selected patients with chronic lateral elbow pain [12]. These studies provide an important foundation for recognizing a superficial sensory nerve component in recalcitrant lateral elbow pain. However, their primary focus has been identification of the PABCN itself or perineural injection around the nerve. In contrast, the present study was designed to test whether a single humerus-parallel lateral elbow window could allow injectate distribution to both the superficial PABCN-related fascial plane and the common extensor origin–lateral epicondylar enthesis complex. Therefore, the novelty of the present study is not sonographic localization of the PABCN, but the cadaveric demonstration of a single enthesis-centered fascial-plane pathway that anatomically encompasses both superficial sensory nerve-related branches and the tendon–enthesis target region.

The cadaveric dye findings support the anatomical plausibility of this dual-region concept. Dye staining of both the anterior and posterior PABCN branches suggests that the injectate reached the superficial nerve-related fascial plane rather than remaining confined to the tendon surface. At the same time, dye coverage over the common extensor aponeurosis and lateral epicondylar enthesis indicates that the same injectate pathway also reached the tendon–enthesis region, which remains the principal structural target in lateral epicondylopathy. These findings should be interpreted as anatomical evidence of target-region accessibility, not as evidence that this approach is clinically superior to isolated tendon-directed or nerve-directed injections. The methodological rationale of this study is consistent with cadaveric injection studies in which ultrasound-guided needle placement is followed by anatomical dissection to confirm whether an intended target or fascial plane can be reached [9,10,13]. Such studies can provide useful anatomical and technical information, particularly during early procedural development. However, they do not establish in vivo hydrodissection mechanics, clinical efficacy, symptom relief, or comparative effectiveness. In the present study, the two elbows were regarded as descriptive procedural replications from a single donor rather than independent biological samples. Accordingly, the findings should be interpreted as a proof-of-concept anatomical demonstration of the proposed pathway.

A potential technical feature of the humerus-parallel longitudinal approach is that it uses a single working window and a continuous in-plane needle trajectory over the common extensor aponeurosis. This configuration may be useful for procedural standardization and education because it relies on recognizable bony and tendinous landmarks. However, because this study did not include a comparative injection arm, procedural time measurement, or needle-pass analysis, no conclusion can be drawn regarding superiority, efficiency, or accuracy compared with conventional PABCN-centered or tendon-directed approaches.

The present study did not evaluate any therapeutic injectate, biologic agent, regenerative intervention, or clinical outcome. Therefore, the findings should be regarded only as hypothesis-generating for future interventional research. If future studies investigate clinical injectates, differences in viscosity, tissue interaction, injection pressure, and biological behavior must be considered. Methylene blue was used as a visual tracer for anatomical mapping and may spread differently from platelet-rich plasma, dextrose-based injectates, local anesthetics, or other clinically used preparations. Therefore, dye distribution in this cadaveric model should not be assumed to predict the exact distribution or therapeutic effect of clinical injectates.

Several potential failure modes should be considered before clinical translation. First, continuity of the superficial fascial plane may vary across individuals, particularly in patients with prior trauma, surgery, scarring, inflammatory change, or advanced degenerative tissue alteration. Second, age-related changes in fascia, subcutaneous tissue, and tendon quality may influence injectate distribution; the present donor was elderly, and the observed pattern may not represent younger or more muscular individuals. Third, a broad fascial-plane injection may cause unintended spread to adjacent soft tissues or may fail to provide selective neural or entheseal targeting. Fourth, although the PABCN is anatomically relevant, a superficial sensory nerve component may not be clinically important in every patient with lateral epicondylopathy. Finally, because cadaveric tissue lacks perfusion, physiologic tissue pressure, pain response, and dynamic muscle tone, the observed dye distribution may differ from in vivo injectate behavior.

Several safety considerations should be emphasized. The PABCN is a superficial sensory nerve, and direct intraneural injection or mechanical contact should be avoided. The goal of the proposed pathway is to expand the surrounding superficial fascial plane under continuous ultrasound visualization, not to puncture the nerve. Slow, low-pressure injection, continuous needle-tip visualization, and careful preprocedural scanning are therefore essential. In clinical practice, color Doppler evaluation should be used to identify superficial vessels or vascular variants along the anticipated needle path, and the trajectory should be modified if a safe path cannot be established. In addition, unintended intra-articular spread, intramuscular deposition, and focal subcutaneous pooling should be avoided. Although a preliminary PABCN-related fascial-plane block may theoretically reduce discomfort during subsequent posterolateral elbow procedures, including intra-articular injection, this possibility remains speculative and requires separate clinical evaluation. This concept is also consistent with previous cadaveric work suggesting that the PABCN may be relevant during posterolateral elbow procedures, including ultrasound-guided intra-articular injection [14,15].

This study has several limitations. First, it was performed using both elbows from a single fresh-frozen, non-embalmed cadaveric donor. Although bilateral consistency was observed, the two elbows were not independent biological samples and cannot establish reproducibility across donors, body habitus, sex, age, tissue quality, or anatomical variants of the PABCN. Second, the study was not designed to assess operator-independent feasibility because all injections were performed by an experienced musculoskeletal ultrasound operator. Third, although the injection operator and anatomist performed their procedures independently and the anatomist was not informed of the detailed needle trajectory or anticipated dye distribution before dissection, this was not a formal blinded quantitative assessment. Fourth, spatial dye spread was measured directly during dissection using a metric ruler and was additionally reviewed using ImageJ software (version 1.54p; National Institutes of Health, Bethesda, MD, USA) for supportive image-assisted assessment of ruler-containing photographs. However, the photographs were obtained for anatomical documentation rather than under a fully standardized quantitative imaging protocol. Therefore, the reported values should be interpreted as descriptive gross linear measurements rather than formal digital segmentation, volumetric quantification, proportional coverage analysis, or dye-intensity analysis. Fifth, histologic validation of the injected fascial plane was not performed. Sixth, methylene blue is a visual tracer and does not replicate the viscosity, diffusion behavior, or tissue-plane dynamics of clinical injectates. Seventh, cadaveric injectate distribution cannot reproduce living tissue conditions, including vascular perfusion, physiologic tissue pressure, pain response, inflammatory change, or dynamic tissue compliance. Finally, this study did not include a comparative injection arm and cannot determine whether the proposed approach is more accurate, efficient, safer, or clinically more effective than existing tendon-directed or PABCN-centered injection techniques.

Future studies should evaluate this approach in larger cadaveric series to determine reproducibility across different donors and anatomical variants. Standardized photographic protocols, blinded assessment, segmentation-based analysis, proportional target coverage, inter-rater reliability testing, and histologic confirmation may strengthen anatomical validation. Paired comparative cadaveric studies could also clarify whether the humerus-parallel pathway differs from conventional PABCN-centered or tendon-directed injections in target coverage, unintended spread, procedure time, or needle redirection. Only after such anatomical validation should clinical studies evaluate diagnostic value, safety, patient selection, symptom response, functional outcomes, and durability of effect.

## 5. Conclusions

This proof-of-concept cadaveric anatomical feasibility study showed that an ultrasound-guided humerus-parallel injectate-distribution pathway could deliver methylene blue to the superficial posterior antebrachial cutaneous nerve (PABCN)-related fascial plane while also covering the common extensor aponeurosis and lateral epicondylar enthesis in both elbows of a single fresh-frozen donor. Direct gross anatomical measurement, supported by calibrated ImageJ review of ruler-containing photographs, demonstrated a descriptive gross linear dye distribution of approximately 10 cm proximally, 5 cm distally, and 4 cm anteriorly from the distal needle entry point. These findings support the anatomical plausibility of a single-window, enthesis-centered dual-region pathway, but they should not be interpreted as evidence of procedural reproducibility, clinical efficacy, safety, or superiority over existing injection techniques. Larger cadaveric studies with standardized photographic protocols, blinded assessment, segmentation-based coverage analysis, and histologic validation are required before clinical translation can be considered.

## Figures and Tables

**Table 1:** Summary of procedural feasibility and anatomical dye distribution after ultrasound-guided humerus-parallel longitudinal injection.

Assessment Category	Item Assessed	Finding	Interpretation
Procedural feasibility	Completion of ultrasound-guided injection	Injection was completed in both elbows using the same standardized protocol	The proposed approach was completed in this cadaveric setting
Procedural feasibility	Sonographic working window	The lateral epicondyle, common extensor aponeurosis, radiocapitellar region, and superficial fascial plane were visualized within the same longitudinal window	The humerus-parallel orientation provided a predefined procedural field.
Procedural feasibility	Needle visualization	The distal-to-proximal in-plane trajectory allowed continuous visualization of the needle shaft and tip	The trajectory supported controlled needle advancement over the common extensor aponeurosis
Real-time injectate behavior	Superficial fascial-plane expansion	Injectate expanded longitudinally along the superficial fascial plane overlying the common extensor origin	The injectate distributed along the intended superficial fascial plane rather than forming a localized subcutaneous bleb
Superficial neural-plane distribution	PABCN-related fascial plane	Methylene blue spread was observed along the superficial fascial plane containing the PABCN	The injectate reached the intended superficial sensory nerve-related plane
Superficial neural-plane distribution	Anterior branch of the PABCN	Dye staining was observed	The anterior branch was included within the dye-stained plane
Superficial neural-plane distribution	Posterior branch of the PABCN	Dye staining was observed	The posterior branch was included within the dye-stained plane
Tendon-enthesis distribution	Common extensor aponeurosis	Superficial dye coverage was confirmed	The injectate reached the tendon surface component of the target region
Tendon-enthesis distribution	Lateral epicondylar enthesis	Dye was observed at the enthesis-centered target region	The injectate reached the common extensor origin-enthesis complex
Spatial extent of dye spread	Proximal spread	Approximately 10 cm from the distal needle entry region	Descriptive evidence of longitudinal continuity toward the distal spiral groove region
Spatial extent of dye spread	Distal spread	Approximately 5 cm from the distal needle entry region	Descriptive evidence of distal fascial-plane spread
Spatial extent of dye spread	Anterior spread	Approximately 4 cm across the lateral elbow soft-tissue plane	Descriptive evidence of anterior extension across the lateral elbow region
Unintended spread	Gross intra-articular dye deposition	Not observed	No gross evidence of unintended intra-articular injection
Unintended spread	Focal intramuscular deposition	Not observed	No gross evidence of focal intramuscular pooling

PABCN, posterior antebrachial cutaneous nerve. Because both elbows were obtained from a single cadaveric donor, the findings were interpreted as descriptive anatomical observations rather than independent biological outcomes. Linear dye-spread distances were measured directly during anatomical dissection using a metric ruler and were additionally reviewed using ImageJ software (version 1.54p; National Institutes of Health, Bethesda, MD, USA) for supportive image-assisted assessment. ImageJ review was used only as supportive assessment of gross linear measurements; no formal segmentation, volumetric quantification, proportional coverage analysis, dye-intensity analysis, histologic validation, or statistical comparison was performed.

## Data Availability

The data supporting the findings of this study are available from the corresponding authors upon reasonable request.

## Supplementary Materials

The following supporting information can be downloaded at: https://www.mdpi.com/article/10.3390/diagnostics16111698/s1, Video S1. Real-time ultrasound-guided humerus-parallel distal-to-proximal in-plane needle advancement and superficial fascial-plane injectate distribution over the common extensor aponeurosis. The video demonstrates the 23-gauge needle introduced from a distal skin entry point and advanced proximally using an in-plane approach while maintaining continuous sonographic visualization of the needle shaft and tip. Slow low-pressure injection demonstrates longitudinal fluid expansion along the superficial fascial plane overlying the common extensor aponeurosis. The video is provided to improve procedural reproducibility and to illustrate the real-time ultrasound-guided component of the proposed approach.

## Abbreviations

AB, anterior branch; BR, brachioradialis; CE, common extensor aponeurosis; DSG, distal spiral groove; ECRB, extensor carpi radialis brevis; ECRL, extensor carpi radialis longus; EDC, extensor digitorum communis; ECU, extensor carpi ulnaris; LE, lateral epicondylar enthesis; PABCN, posterior antebrachial cutaneous nerve; PB, posterior branch; PCNF, posterior cutaneous nerve of the forearm; PRP, platelet-rich plasma.

## References

[1] Waseem M., Nuhmani S., Ram C.S., Sachin Y. (2012). Lateral epicondylitis: A review of the literature. J. Back Musculoskelet. Rehabil..

[2] Coombes B.K., Bisset L., Vicenzino B. (2015). Management of Lateral Elbow Tendinopathy: One Size Does Not Fit All. J. Orthop. Sports Phys. Ther..

[3] Bonczar M., Ostrowski P., Dziedzic M., Kasprzyk M., Obuchowicz R., Zacharias T., Marchewka J., Walocha J., Koziej M. (2023). Evaluation of lateral epicondylopathy, posterior interosseous nerve compression, and plica syndrome as co-existing causes of chronic tennis elbow. Int. Orthop..

[4] Kotnis N.A., Chiavaras M.M., Harish S. (2012). Lateral epicondylitis and beyond: Imaging of lateral elbow pain with clinical-radiologic correlation. Skelet. Radiol..

[5] Starr B.W., Lee D.S., Stern P.J. (2020). Anatomy of the Posterior Antebrachial Cutaneous Nerve, Revisited. J. Hand Surg. Am..

[6] Corke P.J. (2019). Ultrasound-guided posterior antebrachial cutaneous nerve block utilising the ‘fat-filled flat tunnel’: Description of technique and cutaneous sensory block area. Anaesth. Intensive Care.

[7] Maida E., Chiavaras M.M., Jelsing E.J., O’Driscoll S.W., Pawlina W., Smith J. (2017). Sonographic Visualization of the Posterior Cutaneous Nerve of the Forearm: Technique and Validation Using Perineural Injections in a Cadaveric Model. J. Ultrasound Med..

[8] Umapathy S., Miller M., Chen Y.T. (2024). Novel Ultrasound-Guided Radiofrequency Ablation of the Epicondylar Branch of the Posterior Cutaneous Nerve of the Forearm for Recalcitrant Lateral Epicondylosis. Cureus.

[9] Kim S.-H., Lee U.Y., Yoon Y., Hwang J., Lee J., Kim S., Lam K.H., Suryadi T., Suhaimi A. (2026). An Anatomical Cadaveric Demonstration of an Ultrasound-Guided Fascial Plane Injection Pathway in the Deep Gluteal Space. Bioengineering.

[10] Kim S.-H., Lee U.Y., Yoon Y., Hwang J., Kim J., Na Y., Kim S., Lam K.H., de Castro J.C., Suryadi T. (2026). Ultrasound-Guided Targeted Injection to the Anterior Labral–Ligamentous Complex of the Shoulder: A Cadaveric Feasibility Study for Regenerative Therapy. Bioengineering.

[11] García-Martínez J., Miguel-Pérez M., Pérez-Bellmunt A., Ortiz-Miguel S., Viscor G. (2021). The Course of Posterior Antebrachial Cutaneous Nerve: Anatomical and Sonographic Study with a Clinical Implication. Int. J. Environ. Res. Public Health.

[12] Wagle S., Glazebrook K., Moynagh M., Smith J., Sellon J., Skinner J., Morrey M. (2021). Role of ultrasound-guided perineural injection of the posterior antebrachial cutaneous nerve for diagnosis and potential treatment of chronic lateral elbow pain. Skelet. Radiol..

[13] Suhaimi A., Yoon Y., Suryadi T., Lee J., Lim J., Kim J., Hwang J., Kim S.-H., Lee U.-Y., Lam K.H.S. (2026). Ultrasound-Guided, Single-Entry, Dual-Target Hydrodissection for Carpal Tunnel Syndrome: A Technical Note With Cadaveric Validation. Cureus.

[14] Lam K.H.S., Yoon Y., Su D.C.-J., Suryadi T., Suhaimi A., Wu Y.T. (2026). Ultrasound-Guided Hydrodissection for Peripheral Neuropathy: An Evidence-Based Intervention Whose Time Has Finally Come. Cureus.

[15] Ricci V., Mezian K., Chang K.V., Mittal N., Kara M., Naňka O., Özçakar L. (2023). Ultrasound-guided injection of the elbow: Cadaveric description for the proximal to distal approach. PM R.

